# Binding of Tetracyclines to *Acinetobacter baumannii* TetR Involves Two Arginines as Specificity Determinants

**DOI:** 10.3389/fmicb.2021.711158

**Published:** 2021-07-19

**Authors:** Manuela Sumyk, Stephanie Himpich, Wuen Ee Foong, Andrea Herrmann, Klaas M. Pos, Heng-Keat Tam

**Affiliations:** Institute of Biochemistry, Goethe-University Frankfurt, Frankfurt, Germany

**Keywords:** transcription repressor, antibiotic resistance, *Acinetobacter baumannii*, TetR family, tetracycline transporter, tetracycline, tigecycline

## Abstract

*Acinetobacter baumannii* is an important nosocomial pathogen that requires thoughtful consideration in the antibiotic prescription strategy due to its multidrug resistant phenotype. Tetracycline antibiotics have recently been re-administered as part of the combination antimicrobial regimens to treat infections caused by *A. baumannii*. We show that the TetA(G) efflux pump of *A. baumannii* AYE confers resistance to a variety of tetracyclines including the clinically important antibiotics doxycycline and minocycline, but not to tigecycline. Expression of *tetA(G)* gene is regulated by the TetR repressor of *A. baumannii* AYE (AbTetR). Thermal shift binding experiments revealed that AbTetR preferentially binds tetracyclines which carry a O-5H moiety in ring B, whereas tetracyclines with a 7-dimethylamino moiety in ring D are less well-recognized by AbTetR. Confoundingly, tigecycline binds to AbTetR even though it is not transported by TetA(G) efflux pump. Structural analysis of the minocycline-bound AbTetR-Gln116Ala variant suggested that the non-conserved Arg135 interacts with the ring D of minocycline by cation-π interaction, while the invariant Arg104 engages in H-bonding with the O-11H of minocycline. Interestingly, the Arg135Ala variant exhibited a binding preference for tetracyclines with an unmodified ring D. In contrast, the Arg104Ala variant preferred to bind tetracyclines which carry a O-6H moiety in ring C except for tigecycline. We propose that Arg104 and Arg135, which are embedded at the entrance of the AbTetR binding pocket, play important roles in the recognition of tetracyclines, and act as a barrier to prevent the release of tetracycline from its binding pocket upon AbTetR activation. The binding data and crystal structures obtained in this study might provide further insight for the development of new tetracycline antibiotics to evade the specific efflux resistance mechanism deployed by *A. baumannii*.

## Introduction

*Acinetobacter baumannii* is an opportunistic human pathogen, that has been recently classified by the World Health Organization as the most prevalent clinical bacterium, in need for novel antibiotics due to its multidrug resistance (MDR) phenotype (World Health Organisation [WHO], 2017). The intrinsic antibiotic resistance and the propensity to acquire MDR determinants cause a tremendous problem in public health, leading to high morbidity and mortality associated with nosocomial infections (Touchon et al., 2014; Morris et al., 2019). A prominent MDR mechanism employed by *A. baumannii* is the deployment of multidrug efflux pumps that actively remove a variety of antibiotics from the cells across bacterial membranes (Rumbo et al., 2013; Yoon et al., 2015). The gene expression of multidrug efflux pumps is often modulated by various types of transcriptional regulators including LysR-type transcriptional regulators, TetR-type regulators, and two-component transcriptional regulatory systems (Marchand et al., 2004; Coyne et al., 2010; Rosenfeld et al., 2012; Liu et al., 2018).

Tetracyclines are bacteriostatic antibiotics that function through reversible binding to the A site of the 30S ribosomal subunit, thereby inhibiting bacterial protein synthesis (Chopra and Roberts, 2001). Due to their broad spectrum of activity and relatively low cost, tetracyclines are used extensively in human and animal infections. In many countries, tetracyclines are incorporated into livestock feed at subtherapeutic doses as growth promoters for metaphylaxis purposes (Chattopadhyay, 2014; Granados-Chinchilla and Rodríguez, 2017). The misuse of tetracyclines in the poultry sector has led to an increase in acquired tetracycline resistance and these resistance mechanisms are attributed to efflux pumps, inactivating enzymes, ribosomal protection, and/or target modification (Nguyen et al., 2014). Tetracycline resistance genes in bacteria are typically located in mobile plasmids, transposons, conjugative transposons, and integrons, enabling these genes to move between species and into a wide range of genera by conjugation (Chopra and Roberts, 2001). In the late twentieth century, tigecycline has been specifically developed to overcome the emerging efflux-mediated tetracycline resistance (e.g., TetA) in Gram-negative bacteria, and exhibits an increase in antimicrobial potency against clinically important pathogens (Petersen et al., 1999). The enhanced antimicrobial activity of tigecycline compared to other tetracyclines is attributed to its increased binding affinity for the ribosome (Olson et al., 2006). Notably, the bulky 9-*tert*-butyl-glycylamido moiety at position C9 of tigecycline has enabled this antibiotic to escape the TetA-mediated extrusion of tigecycline, most likely due to the steric hindrance effect to TetA caused by this bulky substituent (Hirata et al., 2004).

In Gram-negative bacteria, it became evident that tetracyclines are transported out of the cells in a synergy between the single component efflux pumps (e.g., TetA) and the Resistance Nodulation cell Division (RND)-type tripartite efflux pumps, both energized by the proton motive force (McMurry et al., 1980; Foong et al., 2020). In *A. baumannii*, tetracyclines are initially transported from the cytoplasm to the periplasm by TetA, from where subsequently, RND-type efflux pumps (e.g., AdeABC, AdeFGH, and AdeIJK) remove the antibiotics from the periplasm across the outer membrane (Foong et al., 2020). The expression of *tetA* is tightly regulated by TetR, a member of TetR-family transcriptional regulators (TFR) and *tetA* expression is induced by sub-inhibitory tetracycline concentrations (Takahashi et al., 1986). TFRs harbor a highly variable C-terminal sensory ligand-binding domain (LBD) and a conserved N-terminal DNA-binding domain (DBD) (Ramos et al., 2005; Cuthbertson and Nodwell, 2013). The DBD is composed of three α-helices forming a helix-turn-helix (HTH) motif, and interacts with the DNA major groove (Hinrichs et al., 1994; Orth et al., 2000). The LBD is responsible for ligand binding and oligomerization (Hinrichs et al., 1994; Kisker et al., 1995). In the absence of the ligand, the dimeric TFR repressor binds to a specific operator sequence, preventing the transcription of its target genes. Upon ligand binding, conformational changes of the TFR repressor trigger a pendulum movement of the DBD, thereby leading to the release of repressor from the operator DNA. The dissociation of the TFR repressor from the operator DNA subsequently activates the expression of TFR target genes (Kisker et al., 1995; Orth et al., 2000).

A recent study has indicated that TetA(G) of *A. baumannii* AYE confers resistance to the clinically important tetracycline antibiotics doxycycline and minocycline. Genome sequence analysis of *A. baumannii* AYE revealed the presence of a divergently transcribed TFR gene (ABAYE3639, hereafter referred to as *A. baumannii AbtetR*) located downstream of the *tetA* gene (Fournier et al., 2006). Here, we show that expression of the *A. baumannii tetA(G)* gene in *E. coli* Δ*mdfA*Δ*emrE* confers resistance to various tetracyclines except tigecycline. The *tetR* gene encodes a transcriptional regulator that controls the *tetA(G)* expression. In this study, we found that AbTetR binds to an intercistronic region between *tetA* and *tetA(G)* genes. In addition, thermal shift binding experiments revealed that AbTetR prefers to bind tetracyclines, which carry a O-5H moiety in ring B. In contrast, tetracyclines (e.g., minocycline and tigecycline) with a 7-dimethylamino moiety are less well recognized by AbTetR. Structural analysis of a minocycline bound AbTetR-Gln116Ala variant showed that Arg104 and Arg135, which are embedded at the entrance of the binding pocket, are important for tetracycline recognition and act as a barrier to prevent the release of tetracycline from the AbTetR binding pocket.

## Materials and Methods

### Cloning of *A. baumannii tetA(G)* and *tetR* and Site-Directed Mutagenesis

*A. baumannii tetR* and *tetA(G)* genes were cloned into pET24a and pTTQ18, respectively (Foong et al., 2020), via the Gibson assembly method (Gibson et al., 2009). Briefly, *tetR* and *tetA(G)* were amplified from chromosomal DNA of *A. baumannii* AYE. Amplified genes and vectors were mixed with the Gibson assembly mixture containing T5 exonuclease (Epicenter), Phusion polymerase (Thermo Fisher Scientific) and Taq DNA ligase (NEB). AbTetR substitution variants were generated by the ExSite protocol (Stratagene) with 5′-phosphorylated primers. Plasmids were verified by sequencing (Eurofins Scientific). All primers used in this study are listed in Supplementary Table 1.

### Drug Susceptibility Assay

**FIGURE 1:**
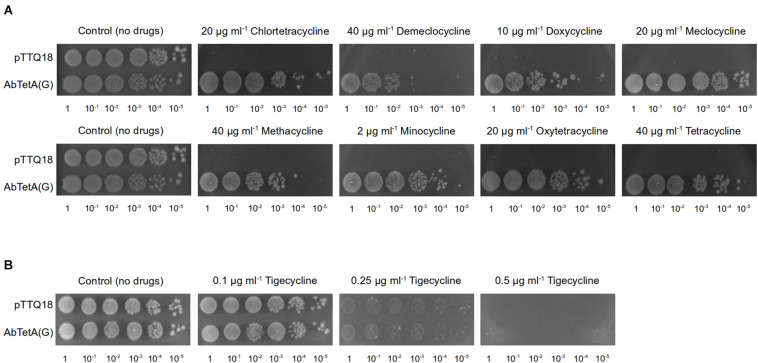
TetA(G) from *A. baumannii* confers resistance to *E. coli* BW25113 Δ*emrE*Δ*mdfA* toward diverse tetracyclines. Drug susceptibility assays were conducted by spotting diluted cell cultures (OD_600_ = 10^0^–10^–5^, as indicated below the respective images) on LB agar plate supplemented with 0.2 mM IPTG and 100 μg ml^–1^ ampicillin containing **(A)** tetracyclines (20 μg ml^–1^ chlortetracycline, 40 μg ml^–1^ demeclocycline, 10 μg ml^–1^ doxycycline, 20 μg ml^–1^ meclocycline, 40 μg ml^–1^ methacycline, 2 μg ml^–1^ minocycline, 20 μg ml^–1^ oxytetracycline, or 40 μg ml^–1^ tetracycline). Both of the control plates are identical and both represent the same biological repeat in the same experiment (a representative). **(B)** tigecycline (0.1–0.5 μg ml^–1^). As a negative control, cells harboring pTTQ18 (empty vector) were spotted on the same agar plate. Experiments were conducted three times (biological repeats) and the results shown are representative.

Drug susceptibility assays were conducted as previously described (Foong et al., 2019). Briefly, overnight cell cultures of *E. coli* BW25113 Δ*emrE*Δ*mdfA* harboring empty vector (pTTQ18) or pTTQ18abtetG were adjusted to an OD_600_ of 10^0^–10^–5^ and 4 μl of the diluted cultures were spotted on a LB agar plate supplemented with 100 μg ml^–1^ ampicillin, 0.2 mM isopropyl-β-D-1-thiogalactopyranoside (IPTG) and a variety of tetracycline antibiotics (Supplementary Figure 1) at the indicated concentrations (Figure 1). Plates were incubated at 37°C overnight.

### Overproduction and Purification of AbTetR

*E. coli* C43 (DE3) Δ*acrAB* harboring pET24abtetR_His_ was grown overnight in LB liquid medium supplemented with 50 μg ml^–1^ kanamycin (LB_Kan_). Two ml of overnight culture was inoculated into fresh LB_Kan_ liquid medium (1 l in a 5 l baffled Erlenmeyer flask), grown at 37°C at 100 rpm until OD_600_ of 0.6–0.7 before induction with 0.3 mM IPTG (final concentration). Subsequently, the culture was incubated at 20°C for 16 h, at 100 rpm. All protein purification steps were conducted at 4°C. Cells were harvested by centrifugation and suspended in ice-cold Buffer A (50 mM Tris, pH8.0, 500 mM NaCl, 20 mM Imidazole, 10% Glycerol) before passage through the chamber of a Pressure Cell Homogeniser (Stansted, United Kingdom) at 15,000 psi. Insoluble debris was removed by centrifugation at 120,000 × *g* for 45 min. The supernatant was loaded onto a HisTrap HP Ni^2+^ affinity column (GE Healthcare) pre-equilibrated with Buffer A. After two wash steps with 50 column volumes of the same buffer supplemented with 30 and 50 mM imidazole, respectively, bound proteins were eluted in the same buffer supplemented with 230 mM imidazole. The eluted proteins were concentrated to 1–2 ml with Amicon Ultra-15 Centrifugal Filter unit (30 kDa MWCO) (Merck). Subsequently, concentrated proteins were subjected to size-exclusion chromatography (Superdex 75 HiLoad 16/60 column, coupled to an Äkta Prime system, GE Healthcare) using Buffer B (20 mM HEPES, pH8.0, 195 mM NaCl, 5 mM KCl, 5 mM DTT, 5% Glycerol) as running buffer at 0.4 ml min^–1^.

### Thermal Shift Assay

Thermo stability of AbTetR was determined using a Rotor Gene-Q cycler (Qiagen, Hilden, Germany) with Sypro Orange dye as an unfolding reporter (Niesen et al., 2007). Briefly, 2 μl of AbTetR (20 μM) was mixed with 22 μl Buffer B. For ligand-induced melting temperature shifts, chlortetracycline, demeclocycline, doxycycline, meclocycline, methacycline, minocycline, oxytetracycline, tetracycline, or tigecycline was added (300 μM final concentration) to the protein solution and incubated at room temperature for 10 min. Samples were subjected to centrifugation at 13,000 × *g* for 5 min at room temperature, to remove any traces of precipitate. Subsequently, samples were mixed with 1.1 μl of 250× Sypro Orange (Invitrogen). Thermal denaturation was induced by increasing the temperature from 25 to 75°C at a rate of 1°C min^–1^. The fluorescence of the dye was monitored (excitation and emission wavelength of 470 and 555 nm, respectively) during the heating process. The unfolding temperature (*T*_m_) was determined by fitting the fluorescence curve to a Boltzmann sigmoid function (GraphPad Prism). The melting curves are shown in Supplementary Figure 2.

### Electrophoretic Mobility Shift Assay

Electrophoretic mobility shift assay (EMSA) was performed with SYBR Green as a DNA binding probe. Primers used for amplification of the intercistronic region with different DNA sequence length used in the EMSA are listed in Supplementary Table 1. Approximately 100 ng of amplified DNA fragments were incubated with 5 μM AbTetR in binding buffer containing 10 mM Tris, pH7.5, 1 mM EDTA, 100 mM KCl, 5 mM dithiothreitol, 5% glycerol, 0.01 mg ml^–1^ BSA, and 70 ng poly [d(I-C)] as a non-specific competitor. The samples were incubated at 25°C for 30 min and subjected to electrophoresis on a 10% non-denaturing polyacrylamide gel in 1× TBE buffer in an ice-bath at 120 V for 2 h. Subsequently, the gel was stained with 1× SYBR Green in 1× TBE buffer at room temperature for 1 h before de-staining with water. The protein-DNA complexes and free DNAs were detected on an ImageQuant LAS 4000 [Excitation with Epi-RGB (Cy2) and emission filter of Y515Di (Cy2)] (GE Healthcare BioSciences AB, Uppsala, Sweden).

### Crystallization of AbTetR

Crystals of AbTetR were obtained by sitting drop vapor diffusion within 1 week by incubation of equal volumes of protein solution (15 mg ml^–1^) and precipitant solution containing 0.1 M Tris, pH8.5, 0.2 M magnesium chloride hexahydrate, 0.2 M sodium sulfate, and 25% PEG2000 MME. Crystals were cryo-protected by soaking in mother liquor supplemented with increasing PEG2000 MME concentration to 35% before flash-cooling in liquid nitrogen. Crystals of minocycline bound AbTetR-Gln116A were obtained by co-crystallization. Briefly, 8 mg ml^–1^ of AbTetR-Gln116Ala (Gln116Ala) variant was incubated with 1 mM minocycline, dissolved in Buffer B (20 mM HEPES, pH8.0, 195 mM NaCl, 5 mM KCl, 5 mM DTT, 5% Glycerol) at room temperature for 10 min. Subsequently, sample was centrifuged at 13,000 × *g* for 5 min at room temperature to remove any precipitates. Crystals of minocycline-Gln116Ala binary complex were obtained by sitting drop vapor diffusion within 3 days by incubation of equal volumes of the minocycline-Gln116Ala complex solution and precipitant solution containing 0.1M sodium cacodylate, pH5.5, 11% PEG Smear Broad (Molecular Dimension), 3% Tacsimate, pH7.0 (Hampton Research), and 10% ethylene glycol. Crystals were flash-cooled in liquid nitrogen directly from the drop without any cryo-protectant.

### X-Ray Diffraction Data Collection, Processing, and Refinement

Datasets were collected on beam line Proxima 2A at the Soleil Synchrotron, Saint-Aubin, France using a Eiger detector (Dectris), and subsequently indexed and integrated with XDS (Kabsch, 2010). A molecular replacement solution for wildtype AbTetR was obtained using MrBUMP (Keegan and Winn, 2008) using a modified structure by Sculptor (Bunkóczi and Read, 2011) of TetR(D) variant (1A6I) (Orth et al., 1998) as a search model. Structural models were built in COOT (Emsley et al., 2010), refined with REFMAC5 (Murshudov et al., 2011) and validated with MolProbity (Chen et al., 2010). 100% of the residues are in favored regions of the Ramachandran plot for both structures reported in this manuscript. Polder electron density maps were calculated using phenix.polder (Liebschner et al., 2017).

### Tetracycline Bound AbTetR Models

The electron density of minocycline in the Gln116Ala structure was used as a template for ligand (chlortetracycline, demeclocycline, doxycycline, meclocycline, methacycline, oxytetracycline, tetracycline, and tigecycline) fitting with Coot (Emsley et al., 2010). Structural models were refined with REFMAC5 (Murshudov et al., 2011). These structural models were used to interpret the thermal shift binding experiments as shown in Figure 7.

## Results

The *A. baumannii tetR* gene is part of a divergently transcribed regulon, comprises the putative tetracycline transporter gene *tetA(G)*, and the putative transcriptional regulator *tetR* (*abtetR*). AbTetR is presumably involved in the regulation of *tetA(G)* expression. To identify all possible ligands of AbTetR, we first analyzed the substrate transport profile of *A. baumannii* TetA(G) in *Escherichia coli*, followed by subsequent biophysical characterization of AbTetR binding to the identified ligands and operator DNA.

### TetA(G) From *A. baumannii* AYE Confers Resistance to Various Tetracyclines

A previous study reported that *E. coli* expressing *A. baumannii tetA(G)* confers resistance to tetracycline, minocycline, and doxycycline (Foong et al., 2020). In addition to the aforementioned tetracyclines, overexpression of *tetA(G)* in *E. coli* exerted a protective effect against the bacteriostatic effect of other tetracyclines such as chlortetracycline, demeclocycline, meclocycline, methacycline, and oxytetracycline (Figure 1A and Supplementary Figure 1). Consistent with previous result (Foong et al., 2020), cells expressing *A. baumannii tetA(G)* were susceptible to tigecycline (Figure 1B and Supplementary Figure 1), indicating tigecycline is not recognized by the AbTetA(G) efflux pump.

### Mapping of the DNA Binding Site of AbTetR

**FIGURE 2:**
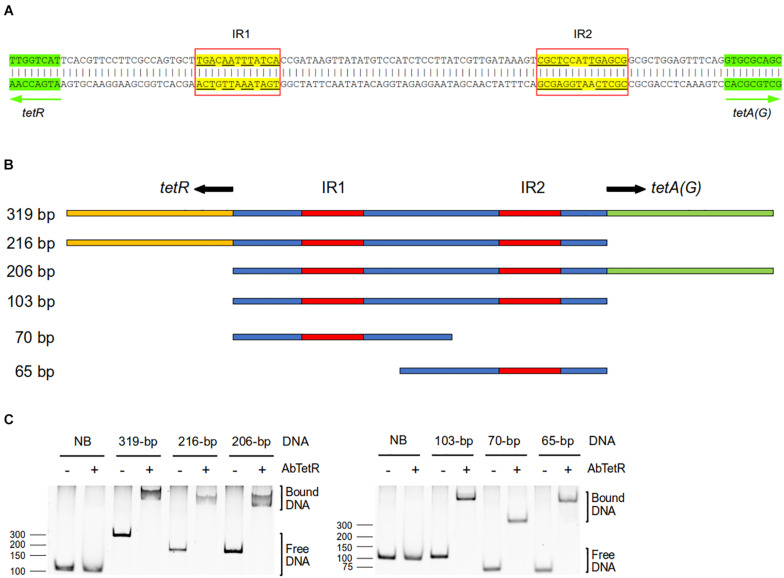
Intercistronic region of *tetR* and *tetA(G)*. **(A)** The *A. baumannii* AYE intercistronic region of *tetR*-*tetA(G)* was analyzed for operator sequences using the EMBOSS palindromes prediction tool (Rice et al., 2000). The intergenic sequence located between *tetR* and *tetA(G)* genes contains two inverted repeat (IR) sequences, IR1 and IR2, highlighted in yellow. The divergently orientated genes *tetR* and *tetA(G)* are indicated by arrows and highlighted in green. **(B)** Schematic representation of the amplified dsDNA of the intercistronic region between *tetR* and *tetA(G)* genes of *A. baumannii* AYE. PCR products of different length consisting IR1 and/or IR2 (319–65 bp) were amplified from genomic DNA of *A. baumannii* AYE. The starts of the open reading frames of the *tetR* and *tetA(G)* genes are indicated in yellow and green, respectively. The intergenic region is indicated in blue and the IRs are highlighted in red. **(C)** Binding of dsDNA containing IR1 and/or IR2 to TetR in the electrophoretic mobility shift assay.

TFRs bind mostly to palindromic inverted repeat (IR) sequences at the promoter region to modulate the expression of their target genes (Orth et al., 1998; Rodikova et al., 2007). In the genome of *A. baumannii* AYE, *tetA(G)* and *tetR* genes are arranged in a divergently orientated direction, and the 103-bp intercistronic sequence between *tetA(G)* and *tetR* contains two IR sequences of 13-bp (designated as IR1) and 14-bp (designated as IR2) in length (Figure 2A). IR1 is located 21-bp upstream of the *tetR* gene and IR2 is located 15-bp upstream of the *tetA(G)* gene (Figure 2A). To determine whether AbTetR binds to this intercistronic sequence, DNA fragments of different lengths containing IR1 and/or IR2 were amplified and these DNA fragments were subjected to EMSA in the presence of purified AbTetR (Figure 2B). All amplified DNA sequences containing the intercistronic region showed an electrophoretic mobility shift of the DNA fragment in the presence of AbTetR, implying that AbTetR binds to the amplified DNA fragments (Figure 2C). In constrast, a lack of AbTetR binding to DNA was observed when AbTetR was incubated with an amplified 96-bp non-binding DNA sequence between the downstream genes ABAYE3642 and ABAYE3644 (Figure 2C). These results indicated that AbTetR binds to the intercistronic region between *tetA* and *tetR* of *A. baumannii* AYE.

### AbTetR Shows Low Affinity Binding for Minocycline and Tigecycline, but High Affinity for Meclocycline

Since AbTetR binds to the intercistronic sequence between *tetA(G)* and *tetR* and potentially modulates the expression of *tetA(G)* (Figure 2C), we tentatively assumed that the substrates of the TetA(G) efflux pump are also substrates of AbTetR. To test this notion, AbTetR was purified and subjected to thermal shift assay (TSA) in the absence or presence of tetracyclines. The *T*_m_ value of the wildtype AbTetR is 45.6°C (Table 1), indicating that AbTetR is less stable in solution compared to TetR(D) (*T*_m_ = 51.8°C) and other globular proteins (Vedadi et al., 2006; Palm et al., 2020). Interestingly, the *T*_m_ value of AbTetR increased to 63.0°C in the presence of tetracycline, with a Δ*T*_m_ [Δ*T*_m_ = *T*_m_(liganded) – *T*_m_(unliganded)] of 17.4°C (Table 1), indicating that tetracycline binds to AbTetR, thereby stabilizing the protein. The pronounced thermostabilization of tetracycline-AbTetR complex is comparable to the Δ*T*_m_ of tetracycline bound TetR(D), with a Δ*T*_m_ of 19.4°C (Palm et al., 2020). In addition to tetracycline, several other tetracyclines stabilized the wildtype AbTetR as well, and caused an apparent increase in *T*_m_ (Table 1). As expected, we observed a significant increase in *T*_m_ of AbTetR in the presence of tigecycline, even though it is not a TetA(G) substrate (Table 1 and Figure 1B). The increase in *T*_m_ of liganded proteins can be correlated to the binding affinity of the ligand (Brandts and Lin, 1990; Matulis et al., 2005). We standardly used tetracycline concentrations of 300 μM in the TSA experiments and therefore, concluded that minocycline and tigecycline are the weakest binders with a Δ*T*_m_ of only 11.5 and 11.0°C, respectively (Table 1). In contrast, the largest increase in Δ*T*_m_ of the liganded AbTetR was obtained in the presence of meclocycline, with an increase of 22.1°C, indicating that meclocycline is the tightest binder. The remaining tetracyclines (chlortetracycline, demeclocycline, doxycycline, methacycline, and oxytetracycline) shifted the Δ*T*_m_ of the liganded AbTetR in the range of 16.7–20.8°C (Table 1 and Supplementary Figure 1). The relative binding affinity of these tetracyclines to AbTetR is minocycline = tigecycline < chlortetracycline = demeclocycline = tetracycline < doxycycline = methacycline = oxytetracycline < meclocycline.

**TABLE 1:** Melting temperatures of AbTetR variants in the absence or presence of various tetracycline antibiotics.

Mutant	Apo	Chl	Δ*T*_m_	ΔΔ*T*_m_	Dem	Δ*T*_m_	ΔΔ*T*_m_	Dox	Δ*T*_m_	ΔΔ*T*_m_	Mec	Δ*T*_m_	ΔΔ*T*_m_	Met	Δ*T*_m_	ΔΔ*T*_m_	Min	Δ*T*_m_	ΔΔ*T*_m_	Oxy	Δ*T*_m_	ΔΔ*T*_m_	Tet	Δ*T*_m_	ΔΔ*T*_m_	Tig*	Δ*T*_m_	ΔΔ*T*_m_
Wildtype	45.6 ± 0.2	62.3± 0.3	16.7	−	62.6 ± 0.1	17.0	−	66.4± 0.2	20.8	−	67.7± 0.3	22.1	−	66.0± 0.3	20.4	−	57.1± 0.3	11.5	−	65.5± 0.3	19.9	−	63.0± 0.3	17.4	−	56.6± 0.3	11.0	−
H64A	n.d.	45.5± 0.4	n.d.	n.d.	46.4± 0.3	n.d.	n.d.	47.7± 0.2	n.d.	n.d.	51.1± 0.5	n.d.	n.d.	47.4± 0.2	n.d.	n.d.	n.d.	n.d.	n.d.	45.6± 0.2	n.d.	n.d.	44.8 ± 0.5	n.d.	n.d.	39.7± 0.9	n.d.	n.d.
N82A	41.5± 0.3	43.5± 0.3	2.0	−14.7	45.7± 0.3	4.2	−12.8	42.8± 0.2	1.3	−19.5	47.1± 0.2	5.6	−16.5	44.1± 0.2	2.6	−17.8	42.4± 0.2	0.9	−10.6	42.4± 0.2	0.9	−19.0	42.3± 0.2	0.8	−16.6	n.d.	n.d.	n.d.
F86A	n.d.	45.1± 0.3	n.d.	n.d.	45.9± 0.1	n.d.	n.d.	49.1± 0.3	n.d.	n.d.	54.2± 0.1	n.d.	n.d.	48.3± 0.2	n.d.	n.d.	n.d.	n.d.	n.d.	46.6± 0.1	n.d.	n.d.	45.6± 0.4	n.d.	n.d.	36.9± 0.7	n.d.	n.d.
H100A	45.9± 0.2	57.3± 0.2	11.4	−5.3	58.7± 0.1	12.8	−4.2	60.9± 0.2	15.0	−5.8	64.8± 0.1	18.9	−3.2	60.8± 0.1	14.9	−5.5	49.1± 0.5	3.2	−8.3	57.0± 0.1	11.1	−8.8	55.4± 0.3	9.5	−7.9	47.7± 0.2	1.8	−9.2
T103A	46.8± 0.1	57.8± 0.2	11.0	−5.7	60.6± 0.3	13.8	−3.2	62.9± 0.2	16.1	−4.7	64.9± 0.2	18.1	−4.0	62.6± 0.3	15.8	−4.6	53.3± 0.2	6.5	−5.0	61.1± 0.3	14.3	−5.6	59.7± 0.3	12.9	−4.5	53.0± 0.2	6.2	−4.8
R104A	40.9± 0.2	**60.6± 0.4**	**19.7**	**3.0**	**60.1± 0.5**	**19.2**	**2.2**	60.9± 0.3	20.0	−0.8	63.1± 0.3	22.2	0.1	61.2± 0.3	20.3	−0.1	52.0± 0.7	11.1	−0.4	**63.4± 0.3**	**22.5**	**2.6**	**60.4± 0.3**	**19.5**	**2.1**	**53.2± 0.4**	**12.3**	**1.3**
Q116A	n.d.	54.4± 0.2	n.d.	n.d.	54.9± 0.1	n.d.	n.d.	59.4± 0.2	n.d.	n.d.	60.2± 0.1	n.d.	n.d.	59.0± 0.1	n.d.	n.d.	49.8± 0.4	n.d.	n.d.	57.1± 0.2	n.d.	n.d.	54.9± 0.3	n.d.	n.d.	44.1± 0.3	n.d.	n.d.
R135A	41.7± 0.2	62.2± 0.2	20.5	3.8	62.7± 0.1	21.0	4.0	**70.1± 0.2**	**28.4**	**7.6**	68.8± 0.1	27.1	5.0	**70.0± 0.2**	**28.3**	**7.9**	57.2± 0.1	15.5	4.0	**67.9± 0.2**	**26.2**	**6.3**	**65.8± 0.2**	**24.1**	**6.7**	56.9± 0.1	15.2	4.2
S138A	42.9± 0.1	59.7± 0.3	16.8	0.1	60.8± 0.1	17.9	0.9	63.4± 0.3	20.5	−0.3	65.7± 0.2	22.8	0.7	62.9± 0.2	20.0	−0.4	53.2± 0.1	10.3	−1.2	62.3± 0.3	19.4	−0.5	60.1± 0.3	17.2	−0.2	53.5± 0.4	10.6	−0.4
E147A	44.8 ± 0.1	56.6 ± 0.2	11.8	−4.9	58.3± 0.1	13.5	−3.5	59.2± 0.1	14.4	−6.4	64.4± 0.1	19.6	−2.5	59.5± 0.1	14.7	−5.7	48.3± 0.2	3.5	−8.0	55.9± 0.2	11.1	−8.8	53.7± 0.2	8.9	−8.5	49.0± 0.4	4.2	−6.8
R104A_ R135A*	n.d.	56.1± 0.2	n.d.	n.d.	54.4± 0.2	n.d.	n.d.	55.5± 0.2	n.d.	n.d.	57.7± 0.4	n.d.	n.d.	57.0± 0.2	n.d.	n.d.	n.d.	n.d.	n.d.	55.5± 0.2	n.d.	n.d.	54.1± 0.1	n.d.	n.d.	53.9± 0.2	n.d.	n.d.

The indicated melting temperature (Tm) were derived using thermal shift assays. Apo, Unliganded; Chl, chlortetracycline; Dem, demeclocycline; Dox, doxycycline; Mec, meclocycline; Met, methacycline; Min, minocycline; Oxy, oxytetracycline; Tet, tetracycline; Tig, tigecycline. Experiments were repeated five times (technical repeats) except for * = three technical repeats. Mean Tm values and standard errors are shown. n.d. = not detected. ΔTm = Tm(liganded) – Tm(unliganded). ΔΔTm = ΔTm(Variant) – ΔTm(Wildtype). The numbers represent in °C as unit. Values in boldface represent T_*m*_ values of the substrate preferences of the respective AbTetR variants.

### Structure of the Unliganded AbTetR and the Minocycline Bound AbTetR-Gln116Ala

**FIGURE 3:**
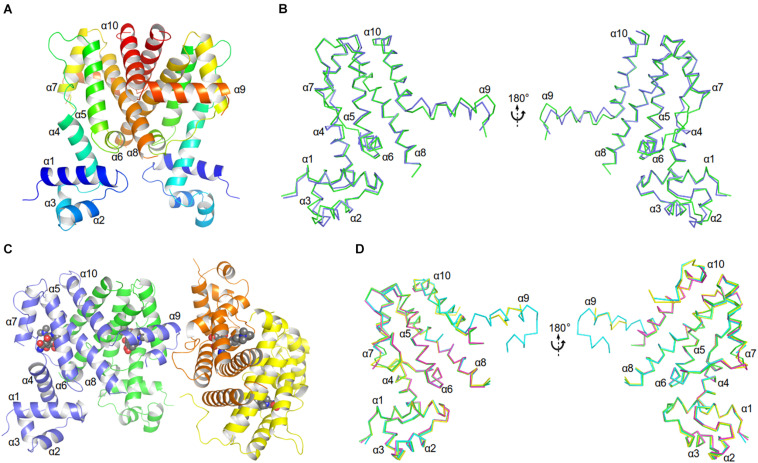
Crystal structures of AbTetR and the minocycline bound AbTetR-Gln116Ala variant. **(A)** Crystal structure of TetR of *A. baumannii* AYE (rainbow color, N terminus: blue, C terminus: red). **(B)** Superimposition of AbTetR-A (green) and AbTetR-B (blue). **(C)** Binary structure of two dimeric Gln116Ala variants in complex with minocycline. Minocycline molecules are depicted as spheres (carbon: black; oxygen: red; nitrogen: blue). **(D)** Superimposition of the four monomers of minocycline-bound Gln116Ala (Gln116Ala-A: green, Gln116Ala-B: cyan, Gln116Ala-C: magenta, Gln116Ala-D: yellow).

The unliganded AbTetR fused to a C-terminal hexahistidine-tag was crystallized in space group *P*2_1_ with two molecules per asymmetric unit, arranging in a twofold rotational symmetry, suggesting a dimer in nature (Figure 3A and Supplementary Table 2). As expected, each of the unliganded AbTetR protomers (Monomer A: AbTetR-A and Monomer B: AbTetR-B) exhibits a typical TFR topology, containing 10 α-helices (Cuthbertson and Nodwell, 2013; Figure 3A), which is well superimposed with other homologous TetR repressors (r.m.s.d. of C_α_ = 1.6–2.4 Å) (Supplementary Figure 3). Both AbTetR protomers are structurally invariant (1.1 Å r.m.s.d. of C_α_ over 176 residues) except for helices α1-α4 and α9, as a result of the involvement of helices α1-4 in crystal packing, whereas helix α9 is highly mobile (Figure 3B). The AbTetR protomer is organized in two core domains, with one core domain being a LDB and the second one being a DBD (Figure 3A). The DBD is composed of helices α1-α3 that form the HTH motif whereas helix α4 connects the DBD and LBD. In contrast, helices α4-α8 form the LBD, whereas helices α8/α8’ and α10/α10’ of the AbTetR-A and AbTetR-B protomers are involved in the formation of a four-helix bundle, thereby contributing to the stabilization of AbTetR dimer.

Extensive crystallization experiments to obtain the crystal structure of the wildtype AbTetR in complex with tetracycline antibiotics were unsuccessful. To obtain AbTetR in complex with tetracyclines, a less active AbTetR variant was used for co-crystallization experiments. Interestingly, the AbTetR-Gln116Ala (Gln116Ala) variant fused to a C-terminal hexahistidine-tag was crystallized in space group *P*2_1_2_1_2_1_ with four molecules per asymmetric unit (Figure 3C and Supplementary Table 2). All the Gln116Ala protomers are invariant (0.45–0.56 Å r.m.s.d. of C_α_ over 168 residues) except for helices α7 and α9, indicating a marginal difference in the asymmetric protomers of the Gln116Ala-AB and Gln116A-CD dimers (discussed later). Similar to the wildtype AbTetR structure, helix α9 of the Gln116Ala variant is likely to be highly mobile even upon minocycline binding, except for the Gln116Ala-B protomer due to the crystal packing (Figure 3D). Minocycline was assigned to the non-proteinaceous electron density in each of the four monomers, and its presence was confirmed by polder electron density map analysis (Liebschner et al., 2017; Figure 3C and Supplementary Figure 4).

**FIGURE 4:**
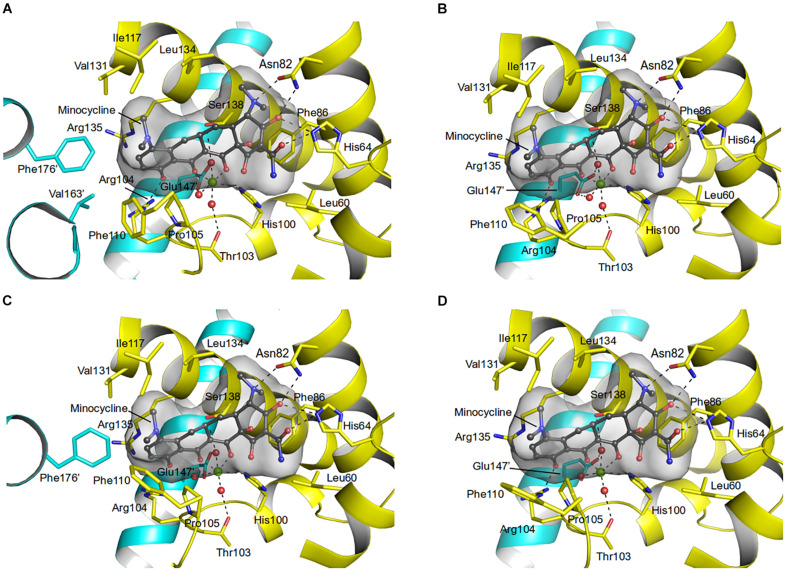
Minocycline binding sites of the AbTetR-Gln116Ala variant. Minocycline bound to **(A)** Monomer A; **(B)** Monomer B; **(C)** Monomer C; and **(D)** Monomer D. Residues from the symmetry-related protomer are colored in cyan. Water molecules and the Mg^2+^ are depicted as red and green spheres, respectively.

**FIGURE 5:**
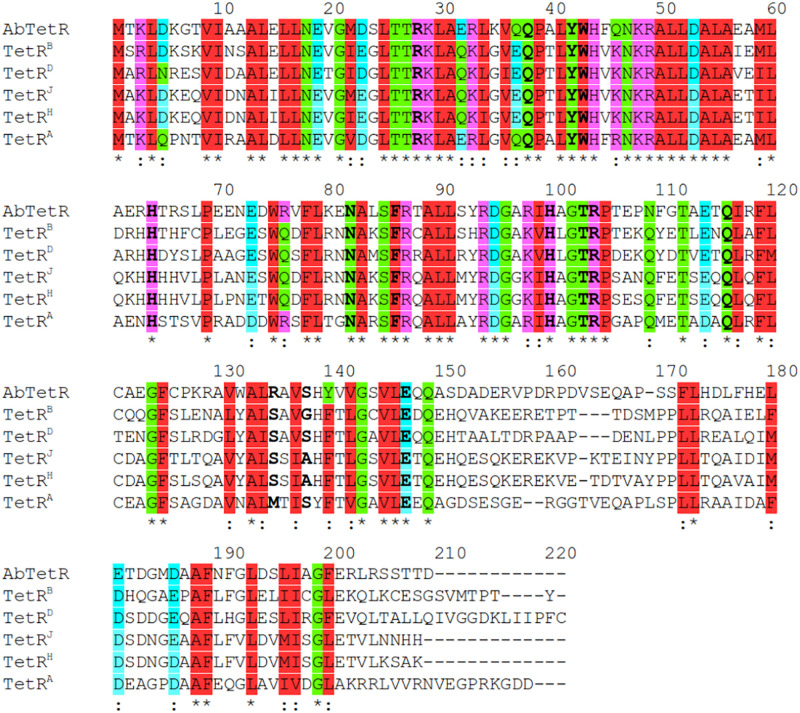
Protein sequence alignment of TetR members. AbTetR: TetR class G of *A. baumannii* AYE (Genbank accession number: CAM88408); TetR^B^: TetR class B of transposon Tn*10* (Genbank accession number: ELD20529); TetR^D^: TetR class D of *Escherichia coli* (Genbank accession number: P0ACT4); TetR^J^: TetR class J from *Proteus mirabilis* (Genbank accession number: AAD12754); TetR^H^: TetR class H from *Pasteurella multocida* (Genbank accession number: CAA75662); TetR^A^: TetR class A from *Pseudomonas* sp. (PDB: 5MRU). Substituted residues are indicated in bold.

Residues (His64, Asn82, Phe86, His100, Thr103, Arg104, Gln116, and Glu147) involved in the tetracycline binding are highly conserved among TetR regulators (Hinrichs et al., 1994; Orth et al., 1998; Figures 4, 5 and Supplementary Figure 5). Similar to the liganded TetR(D) (Hinrichs et al., 1994), the minocycline binding site of AbTetR is defined by helices α4-α6 and α8 (Figure 3C). The entrance of the AbTetR binding pocket consists of helices α7 and α8 from one protomer, and helix α9’ and loop α8’/α9’ from the other protomer (its symmetry-related protomer). Ring A of minocycline engages in hydrogen bond (H-bond) interactions with His64 and Asn82 (Figure 4 and Supplementary Figure 5). Additionally, the O-12aH moiety of minocycline interacts with the phenyl side chain of Phe86 via OH⋯π interaction. In contrast, Leu134 contributes to the van der Waals interaction with the 4-dimethylamino moiety of ring A. A common feature of TetR members is the coordination of the tetracycline-Mg^2+^ complex in the binding pocket via a H-bond network (Takahashi et al., 1986; Hinrichs et al., 1994). In the minocycline bound Gln116Ala structure, Mg^2+^ is coordinated in an octahedral fashion by the keto-enolate group O-11/O-12 of minocycline, His100, and three water molecules (Figure 4). These water molecules form a H-bond network with Thr103, Ser138, and Glu147’ (residue from the symmetry-related protomer). Of note, the involvement of Ser138 in this H-bond network is novel in the minocycline-bound AbTetR structure and this interaction is absent in other TetR regulators (Hinrichs et al., 1994; Kisker et al., 1995; Figures 4, 5 and Supplementary Figure 5). Ring D of minocycline is surrounded by hydrophobic residues (Pro105, Phe110, Ala113, Val131, Ile134, Val163’, and Phe176’), where it is sandwiched between Pro105 and Arg135 by hydrophobic and cation-π stacking interactions, respectively (Figure 4). Notably, Arg135 is not conserved among the TetR-type repressors and this residue is replaced by either serine or methionine in the other TetR repressors (Figure 5 and Supplementary Figure 5). Interestingly, the cation-π stacking interaction between Arg135 and ring D of the tetracycline antibiotics is substituted by a hydrophobic interaction in TetR(B) (Phe177’) and TetR(D) (Met177’) structures (Hinrichs et al., 1994; Supplementary Figure 5). Additionally, the O-10H moiety of minocycline forms a H-bond with Nε of Arg104 in the Gln116Ala structure, but notably this interaction is absent in the other TetR repressors in complex with minocycline (Figure 4 and Supplementary Figure 5).

### Conformational Changes of AbTetR and the Minocycline Bound State

**FIGURE 6:**
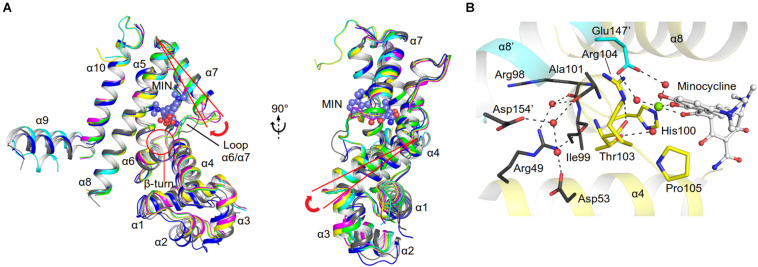
Minocycline (MIN) binding induced conformational changes of AbTetR. **(A)** Conformational changes of the DNA binding domain of the unliganded AbTetR and the liganded Gln116Ala structures. Structural superimposition was performed based on the main chain atoms of helices α8 and α10 (Yu et al., 2010). (AbTetR-A: black, AbTetR-B: blue, Gln116Ala-A: green, Gln116Ala-B: cyan, Gln116Ala-C: magenta, Gln116Ala-D: yellow). **(B)** Water-mediated stabilization of the β-turn upon minocycline binding. Water molecules and the Mg^2+^ are depicted as red and green spheres, respectively.

As discussed above, both protomers of the unliganded AbTetR are virtually indistinguishable except for helices α1-4 and α9 (Figure 3B). Helix α4 of the AbTetR-A protomer is elongated by one helical turn (residues 61–65) in comparison to helix α4 of the AbTetR-B protomer (Figure 3B). Surprisingly, helix α4 of the AbTetR-B protomer adopts a similar conformation akin to the helix α4 of the minocycline bound Gln116Ala structure (Figure 6A). Due to one additional helical turn in helix α4 of the AbTetR-A protomer, Arg63 on helix α4 flips 180° from the solvent exposed position to occupy the position where His64 is located in the liganded state or in the unliganded AbTetR-B protomer, thereby forming H-bonds with Asn82 and Ser138 (Supplementary Figure 6). In addition to the aforementioned H-bond network, Arg63 also engages in a cation-π stacking interaction with Phe86 (Supplementary Figure 6). These unique features enable His64 to adopt a more solvent exposed conformation compared to His64 in the minocycline-bound state. However, we cannot rule out the possibility that this conformation is an artifact due to the interaction between AbTetR-A protomer and its neighboring symmetry protomer.

**FIGURE 7:**
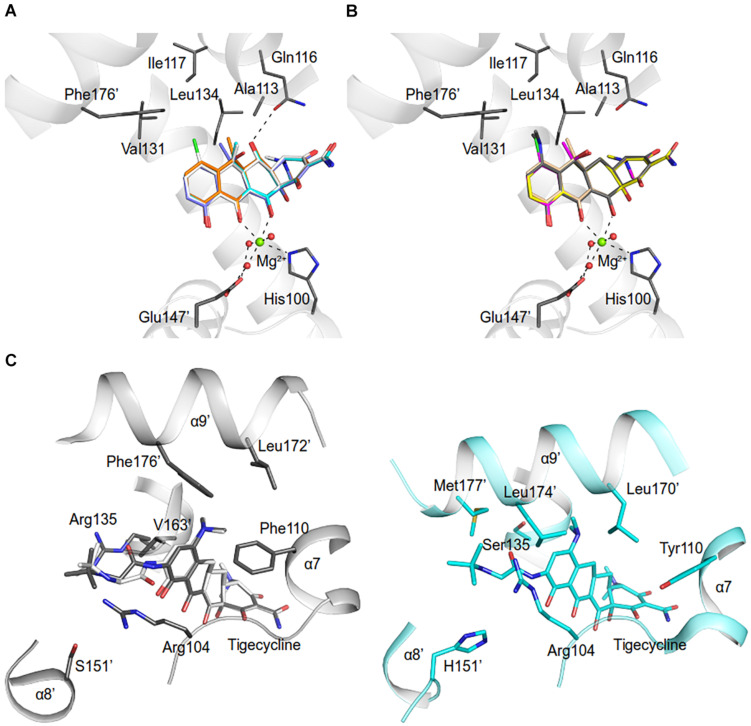
Model of tetracycline antibiotics in the AbTetR binding pocket. Tetracycline antibiotics and Q116 were modeled to the AbTetR structure. Only residues (except for H100 and E147’) that are involved in the interaction with C5, C6, and C7 of tetracyclines are shown as black sticks. Water molecules are depicted as red spheres. Tetracyclines are shown as sticks **(A)** Carbon colored as orange: doxycycline; white: meclocycline; cyan: methacycline; slate: oxytetracycline. **(B)** Carbon colored as magenta: chlortetracycline; yellow: demeclocycline; gray: minocycline; and wheat: tetracycline. **(C)** Tigecycline binding site of TetR(D) (PDB: 4ABZ) (right) and AbTetR (left). Two tigecycline binding modes were modeled to the AbTetR structure. For clarity, only residues that are involved in the interaction with ring D of tigecycline are depicted.

Binding of minocycline to AbTetR appears to induce subtle conformational changes in helix α6, which is comprised of the conserved residues His100–Thr103, forming a type II β-turn (Figure 6A). Notably, the type II β-turn is a typical feature of the induced state in the liganded TetR regulators (Hinrichs et al., 1994; Kisker et al., 1995; Werten et al., 2014). The formation of the β-turn in TetR(D) is mainly attributed to the H-bond network between the Thr103 side chain, the CO moiety of His100, and the tetracycline/Mg^2+^ complex (Orth et al., 1998; Werten et al., 2016). Of note, it has been reported that Thr103 is the key residue in the TetR induction event (Scholz et al., 2000; Werten et al., 2016). Additionally, the β-turn formation in TetR(D) is further stabilized by the salt bridge interaction between Arg104 and Asp178’ (Glu180’ in AbTetR) and the H-bond interaction between Gly102 (conserved in AbTetR) and His151’ (Ser151’ in AbTetR) (Orth et al., 1998). Surprisingly, the β-turn of the minocycline bound Gln116Ala structure is stabilized by a complex H-bond network comprised of Arg49, Asp53, Arg98, Ile99, Ala101, Thr103, Arg104, Asp154’, the minocycline/Mg^2+^ complex and water molecules (Figure 6B). A closer inspection of the AbTetR binding pocket indicated that the β-turn induces a rotation of helix α7 by ∼7.1–8.2°, which is associated with a rotation of helix α4 by 9.9–11.4°, thereby facilitating a pendulum movement of the DBD (Figure 6A). Interestingly, the β-turn formation in the liganded Gln116Ala structure also induces a conformational change of loop α6/α7, which is highly mobile in the unliganded state. Conformational changes of loop α6/α7 include, (i) the formation of one additional helical turn in helix α7 of the Gln116Ala-A protomer; (ii) a poorly defined loop in Gln116Ala-B protomer; and (iii) the formation of a 3_10_ helix in Gln116Ala-C protomer. Rigid body superimposition of the unliganded AbTetR dimer and the minocycline bound Gln116Ala dimer (Gln116Ala-AB, Gln116Ala-CD, and vice versa) revealed a more compact protein folding in the liganded state (Supplementary Figure 7). Binding of minocycline to one liganded protomer, such as the Gln116Ala-A protomer of the Gln116Ala-AB dimer or vice versa (similar to the Gln116Ala-CD dimer or vice versa), leads to a rotation of helices α7’/α8’ in the Gln116Ala-B protomer, resulting in the movement of these helices toward the binding pocket of the Gln116Ala-B protomer (Supplementary Figure 7). The aforementioned motion is followed by a rotation of helices α9’10’ in the Gln116Ala-B protomer toward the binding pocket of the Gln116Ala-A protomer. Taken together, the unique motions of helices α7–α10 in the Gln116Ala-A and Gln116Ala-B protomers trigger a rotation of helices α5/α5’ in the Gln116Ala-AB dimer, inducing pendulum-like movements of helices α4/α4’ and the DBDs in the AbTetR dimer, thereby displacing the AbTetR regulator from the operator DNA (Figure 6A and Supplementary Figure 7).

### Molecular Determinants for Tetracyclines Binding to AbTetR

To elucidate the role of residues embedded in the minocycline binding site, we characterized single alanine AbTetR substitution variants by TSA analysis in the presence of tetracycline antibiotics (Table 1). The unliganded Arg104Ala and Arg135Ala variants exhibited a slightly lower thermal stability than the wildtype AbTetR (*T*_m_ of Arg104Ala = 40.9°C; *T*_m_ of Arg135Ala = 41.7°C) (Table 1). Interestingly, TSA experiments clearly indicated that the ΔΔ*T*_m_ [ΔΔ*T*_m_ = Δ*T*_m_(variant) – Δ*T*_m_(wildtype)] of the Arg104Ala variant under the treatment of chlortetracycline, demeclocycline, oxytetracycline, tetracycline, and tigecycline (between +2.1 and +3.0°C) was higher than the same protein under the treatment of doxycycline, meclocycline, methacycline, and minocycline (between −0.8 and +0.1°C) (Table 1). These results indicate that the Arg104Ala variant exhibits a preference for tetracyclines with a 6-OH substituent except for tigecycline (Table 1 and Supplementary Figure 1). To our surprise, the thermal stability of the Arg135Ala variant increased by 2.4–4.0°C in the presence of doxycycline, methacycline, oxytetracycline, and tetracycline compared to the wildtype AbTetR incubated with the same tetracyclines (Table 1). The thermal stability of the liganded form of the Arg104Ala_Arg135Ala variant was reduced by 2.7–10.9°C in comparison to the wildtype AbTetR upon treatment with tetracyclines, in all cases except for minocycline (Table 1). These results indicate that all tetracyclines bind to the Arg104Ala_Arg135Ala variant except for minocycline. In contrast, the single arginine substitution variants were able to bind minocycline (Table 1).

It has been reported that the TetR(D) substitution variants (His100Ala, Thr103Ala, and Glu147Ala) are more stable than the wildtype protein and binding of tetracycline to these variants increases the thermostability of these proteins by 3.0–8.2°C (Palm et al., 2020). However, the binding affinity of these TetR(D) substitution variants for tetracycline is lower than the binding affinity of the wildtype protein (Palm et al., 2020). Surprisingly, the unliganded AbTetR His100Ala, Thr103Ala, and Glu147Ala variants exhibited comparable *T*_m_ values to the unliganded wildtype AbTetR. As expected, our binding data suggest a slight decrease in the binding affinities of His100Ala, Thr103Ala, and Glu147Ala variants for all tetracyclines except for meclocycline (Table 1). These data are in line with previous reports that His100, Thr103, and Glu147 play a role in tetracycline binding (Palm et al., 2020). Interestingly, the thermostability of the unliganded Ser138Ala variant (*T*_m_ of 42.9°C) was slightly lower compared to the unliganded wildtype AbTetR (*T*_m_ of 45.6°C) (Table 1). However, addition of tetracyclines to the Ser138Ala variant further stabilized this variant, leading to *T*_m_ values similar to the liganded wildtype AbTetR. These results indicate that Ser138 is not important for the coordination of the tetracycline-Mg^2+^ complex. In the minocycline bound Gln116Ala structure, Asn82 is involved in extensive H-bonding with the 4-dimethylamino and 3-enolate moieties of minocycline (Figure 4 and Supplementary Figures 1, 5). Surprisingly, the unliganded Asn82Ala variant exhibited a lower *T*_m_ value (41.5°C) compared to the wildtype AbTetR (Table 1). Previous study indicated that tetracycline binding thermostabilizes the TetR(D)-Asn82Ala variant, leading to a marginal increase in *T*_m_ value (Δ*T*_m_ = 2.2°C) (Palm et al., 2020). As expected, tetracyclines except for demeclocycline, and meclocycline, did not show a pronounced stabilization effect for the AbTetR-Asn82Ala variant, with *T*_m_ values of 42.3–44.1°C (Table 1). Interestingly, incubation of Asn82Ala variant with demeclocycline and meclocycline increased the Δ*T*_m_ value by 4.2 and 5.6°C, respectively. His64Ala, Phe86Ala, and Gln116Ala variants were unstable in solution at temperature ≥ 25°C when compared to other AbTetR variants (Table 1), but addition of tetracyclines stabilized these variants to a certain extent. Interestingly, no clear protein unfolding event was observed for the His64Ala and Phe86Ala variants when these variants were incubated with minocycline. These results indicate that minocycline is not able to bind to the His64Ala and Phe86Ala variants. Moreover, tetracyclines stabilized the Phe86Ala variant less well compared to all the other liganded AbTetR variants (*T*_m_ = 36.9–54.2°C), except for the Asn82Ala variant. Taken together, these results indicate that His64, Asn82, and Phe86 are the most important residues for tetracycline binding. In contrast, Arg104 and Arg135 play a role in tetracycline selectivity.

## Discussion

The binding affinity of tetracyclines to AbTetR is mainly determined by the chemical properties of the tetracycline antibiotics (Table 1, Figure 7, and Supplementary Figure 1). The 7-dimethylamino moiety in ring D is only present in the weakest binders (minocycline and tigecycline) (Table 1 and Supplementary Figure 1). In addition, both minocycline and tigecycline do not have a functional group at position 6 in ring C, whereas the other tetracyclines carry either a methyl, methylene or hydroxyl moiety at this position (Figure 7 and Supplementary Figure 1). Interestingly, addition of the bulky 9-*tert*-butyl-glycylamido moiety in ring D of tigecycline does not affect its binding affinity for AbTetR, as shown by similar *T*_m_ values of the minocycline and tigecycline bound AbTetR complexes (Table 1 and Supplementary Figure 1). Binding of tigecycline to TetR is not unprecedented. In fact, it has been shown experimentally that *tetB* expression is induced by the binding of tigecycline to TetR in *E. coli* (Hirata et al., 2004).

It has been shown that Arg104 plays an important role in the binding of tetracyclines to TetR (Müller et al., 1995). However, our data suggest that Arg104 plays an important role in tetracycline selectivity (Table 1). Interestingly, the Arg104Ala variant exhibits a clear preference for tigecycline and tetracycline antibiotics containing a O-6H moiety in ring C (Table 1 and Supplementary Figure 1). We speculate that substitution of Arg104 to alanine might augment the size of the AbTetR binding pocket associated with alleviating the steric hindrance between the 9-*tert*-butyl-glycylamido moiety of tigecycline and the guanidinium moiety of Arg104, thereby increasing the binding affinity for tigecycline (Figure 7C and Supplementary Figure 1). In contrast, the binding affinity for tigecycline is not affected by the substitution of Arg135 to alanine (Table 1). Our results show that the non-conserved Arg135 is involved in the cation-π stacking interaction with the ring D of minocycline (Figures 4, 5 and Supplementary Figure 5). To our surprise, the Arg135Ala variant shows an increased binding affinity for tetracyclines devoid of a functional moiety at position 7 (Table 1 and Supplementary Figure 1). We therefore speculate that the removal of the Arg135 guanidinium group might create a different local environment in the binding pocket associated with the alteration of binding geometry for tetracyclines compared to the wildtype AbTetR.

One common feature of the tight binding of tetracyclines (doxycycline, oxytetracycline, methacycline, and meclocycline) to AbTetR is the presence of the O-5H moiety in ring B. Indeed, Gln116 putatively interacts with O-5H by H-bonding, as shown by our docking model, thereby increasing the stability of the protein-ligand complexes (Figures 7A,B). The major difference between meclocycline and the other tight binders is the 7-chloro moiety (Supplementary Figure 1). However, the 7-chloro moiety alone cannot be ascribed to an increase in binding affinity for meclocycline, since both chlortetracycline and demeclocycline only show a moderate increase in *T*_m_ (Table 1 and Supplementary Figure 1). Therefore, the O-5H moiety in ring B of meclocycline and doxycycline (absent in chlortetracycline and demeclocycline) plays a crucial role in the binding of these tetracyclines to AbTetR (Supplementary Figure 1). We speculate that the H-bond interaction between the O-5H moiety and Gln116 might further improve the positioning of the meclocycline 7-chloro moiety in the hydrophobic environment, which is comprised of Phe110, Ala113, Ile117, Val131, and Phe176’, thereby contributing to the overall stability of meclocycline in the AbTetR binding pocket (Figures 7A,B).

His64, Asn82 and Phe86 of AbTetR are key residues in the binding of tetracycline antibiotics (Table 1), which corroborates the data from binding studies of TetR(D) (Müller et al., 1995; Scholz et al., 2000; Palm et al., 2020). According to previous studies (Werten et al., 2016; Palm et al., 2020), Asn82 is the most important residue for tetracycline binding that contributes most of the binding energy for the formation of the tetracycline-Mg^2+^ complex in TetR(D) (Palm et al., 2020). Confoundingly, only one tetracycline molecule is identified to bind to one of the two binding pockets in the dimeric TetR(D)-Asn82Ala structure, while the other binding pocket is occupied by an irrelevant molecule (Werten et al., 2016). In contrast, our data suggest that four different tetracyclines (chlortetracycline, methacycline, demeclocycline, and meclocycline) are able to bind to the Asn82Ala variant, as indicated by a slight increase in Δ*T*_m_ value of the protein-ligand complexes (Table 1). Finally, the decrease in thermal stability of the liganded Gln116Ala variant is most likely attributed to the lack of H-bonding between Gln116 and ring A of tetracyclines (Hinrichs et al., 1994; Figures 7A,B and Supplementary Figures 1, 5).

In the unliganded state, loop α6/7, loop α8/9 and helix α9 of AbTetR are highly mobile in order to facilitate the binding of minocycline. As expected, binding of the minocycline-Mg^2+^ complex to AbTetR facilitates the formation of a type II β-turn associated with a rotation of helix α7 toward the minocycline binding pocket, thereby inducing a rigid body motion of helices α8-α10 from its symmetry-related protomer (Supplementary Figure 7). Conformational changes of helices α8 and α8’ in the AbTetR dimer lead to the coordination of Mg^2+^ by Glu147’ in a water-mediated manner, resulting in a rigid body movement of helices α9-α10, α8’/α9’, and loops α8/α9, thereby closing the binding pocket as well as preventing the release of minocycline (Supplementary Figure 7). Surprisingly, binding of minocycline to the Gln116Ala-B and Gln116Ala-D protomers does not induce a complete closure of the binding pocket by helix α9’ from their respective symmetry-related protomer (Figures 4B,D). The partial closure of the binding pocket by helix α9’ is unusual as helix α9’ of the TetR(D) is required to prevent the release of tetracycline from its binding pocket upon TetR(D) induction (Orth et al., 1998). A closer inspection of the AbTetR binding pocket indicated that a complete closure of helices α9/α9’ is not necessary in the AbTetR dimer when minocycline is bound. We speculate that Arg104 and Arg135 embedded at the entrance of AbTetR binding pocket might play an important role as a barrier, together with Pro105, Phe110, and Val131, forming hydrophobic and cation-π traps to prevent the release of minocycline from its binding pocket (Figures 4B,D and Supplementary Figure 5). Additionally, we also speculate that helix α9 plays a role in the retention of minocycline in the binding pocket. Taken together, we propose that the release of AbTetR from its cognate DNA is attributed to cooperativity between two protomers in the AbTetR dimer upon AbTetR induction by tetracycline/Mg^2+^ binding. This cooperativity is mediated by a conformational change of the LDB (helices α7-α10 and α7’-α10’) associated with a pendulum-like motion of helices α4/α4’ and both DBDs, resulting in the release of AbTetR from its cognate DNA (Figure 6A and Supplementary Figure 7).

Tetracyclines are not commonly prescribed to treat the infections caused by *A. baumannii*, however, doxycycline and minocycline have been recently administered in combination with other antibiotics to improve clinical effectiveness in eradicating *A. baumannii* infections (Falagas et al., 2015). It has recently been shown that AbTetA(G) confers resistance to clinically important doxycycline and minocycline (Foong et al., 2020). In fact, our results show that AbTetA(G) exhibits resistance to almost all of the tetracycline antibiotics except for tigecycline (Figure 1). AbTetR, a transcriptional regulator involved in *tetA(G)* expression, can bind various types of tetracycline antibiotics with different binding affinities (Table 1). Interestingly, we show that tigecycline can bind to AbTetR, even though TetA(G) is not able to recognize and transport tigecycline. This result is in line with previous data that *tetB* expression is induced by tigecycline through tigecycline binding to TetR (Hirata et al., 2004). Therefore, we speculate that tigecycline binds to AbTetR, which would render the release of DNA from the repressors, inducing the expression of *tetA(G)*.

The *tetR* and *tetA(G)* genes are embedded within the Tn*7*-like AbaR1 resistance island (Fournier et al., 2006; Rose, 2010). Interestingly, the Tn*7*-like AbaR1 resistance island shows a high similarity to the mobilizable *Salmonella* genomic island 1 of *Proteus mirabilis*, which harbors an antibiotic resistance gene cluster (Siebor and Neuwirth, 2013). It was recently shown that the genomic island 1 is acquired from *Salmonella* spp. (Boyd et al., 2001; Siebor and Neuwirth, 2013), indicating that *A. baumannii* might have acquired the Tn*7*-like AbaR1 resistance island from these species. Alarmingly, it has been demonstrated that the TetA efflux pump confers resistance against tigecycline in *A. baumannii* (Foong et al., 2020). A potential event of horizontal gene transfer between tetracycline susceptible strains and resistant strains, together with a natural selection of tetracycline efflux pumps on tigecycline and an unbridled expression of these genes might pose a serious threat in hospitals. Therefore, the administration of tetracyclines in hospitals has to be carried out with high precaution.

## Data Availability Statement

Atomic coordinates and structure factors for the reported crystal structures have been deposited with the Protein Data Bank under accession number 6RX9 (unliganded AbTetR) and 6RXB (AbTetR-Gln116Ala in complex with minocycline).

## Author Contributions

H-KT conceptualized the work and performed structural analysis. MS, SH, WF, and H-KT conducted all experiments with assistance from AH. H-KT, WF, and KP wrote the manuscript. All authors contributed to the article and approved the submitted version.

## Conflict of Interest

The authors declare that the research was conducted in the absence of any commercial or financial relationships that could be construed as a potential conflict of interest.
