# Supplementary material for: Binding of Tetracyclines to Acinetobacter baumannii TetR Involves Two Arginines as Specificity Determinants
Source: Front Microbiol. 2021 Jul 19;12:711158. doi: 10.3389/fmicb.2021.711158 (PMC8326586; doi:10.3389/fmicb.2021.711158)
Supplement: Supplementary file 1 [file Data_Sheet_1.pdf]

Supplementary Material

Binding of tetracyclines to *Acinetobacter baumannii* TetR involves two arginines as specificity determinants

Manuela Sumyk[1,†], Stephanie Himpich[1,†], Wuen Ee Foong[1], Andrea Herrmann[1], Klaas M. Pos[1], Heng-Keat Tam[1,†,*,§]

[1]Institute of Biochemistry, Goethe-University Frankfurt, Max-von-Laue-Str. 9, D-60438 Frankfurt am Main, Germany.

[†]These authors have contributed equally to this work and share first authorship.

*** Correspondence:**

Heng-Keat Tam

tamhk60@hotmail.com

[§] **Present address:** Hengyang Medical College, University of South China, Hengyang 421002, Hunan Province, China.

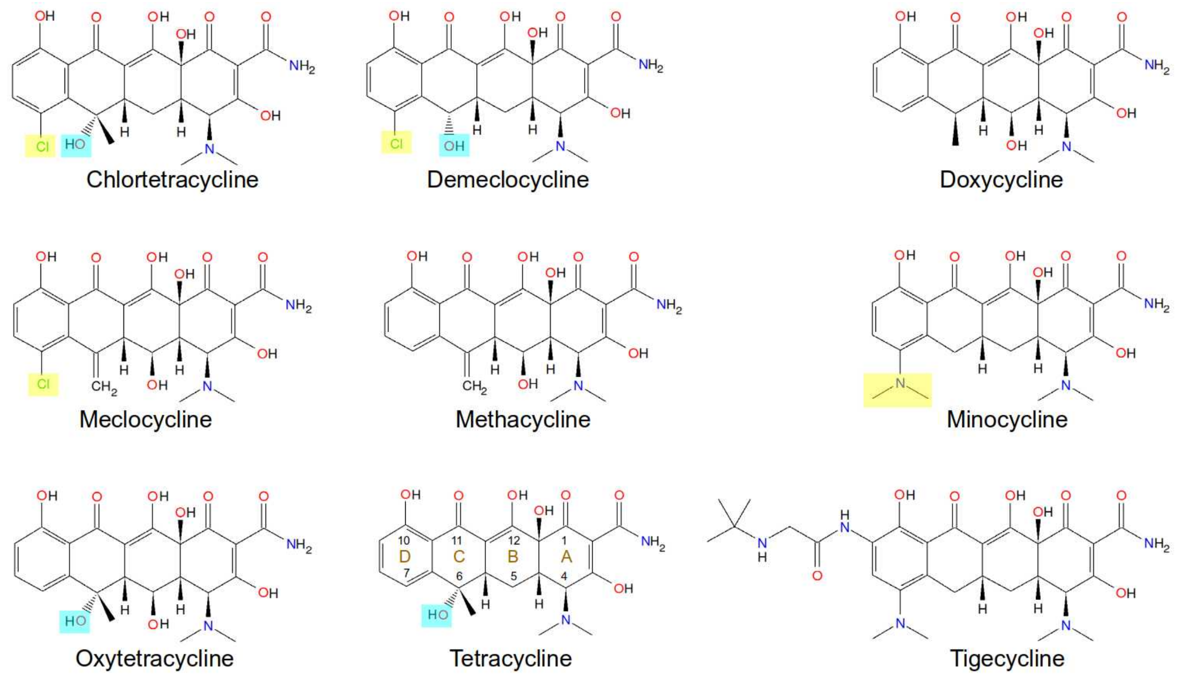

Supplementary Figure 1. Chemical structures of tetracycline derivatives. Tetracyclic core structure and numbering of tetracyclines is indicated at the tetracycline structure. The O-6H and functional groups at position 7 that affect the binding affinities of tetracyclines to Arg104Ala and Arg135Ala are highlighted in cyan and yellow, respectively.

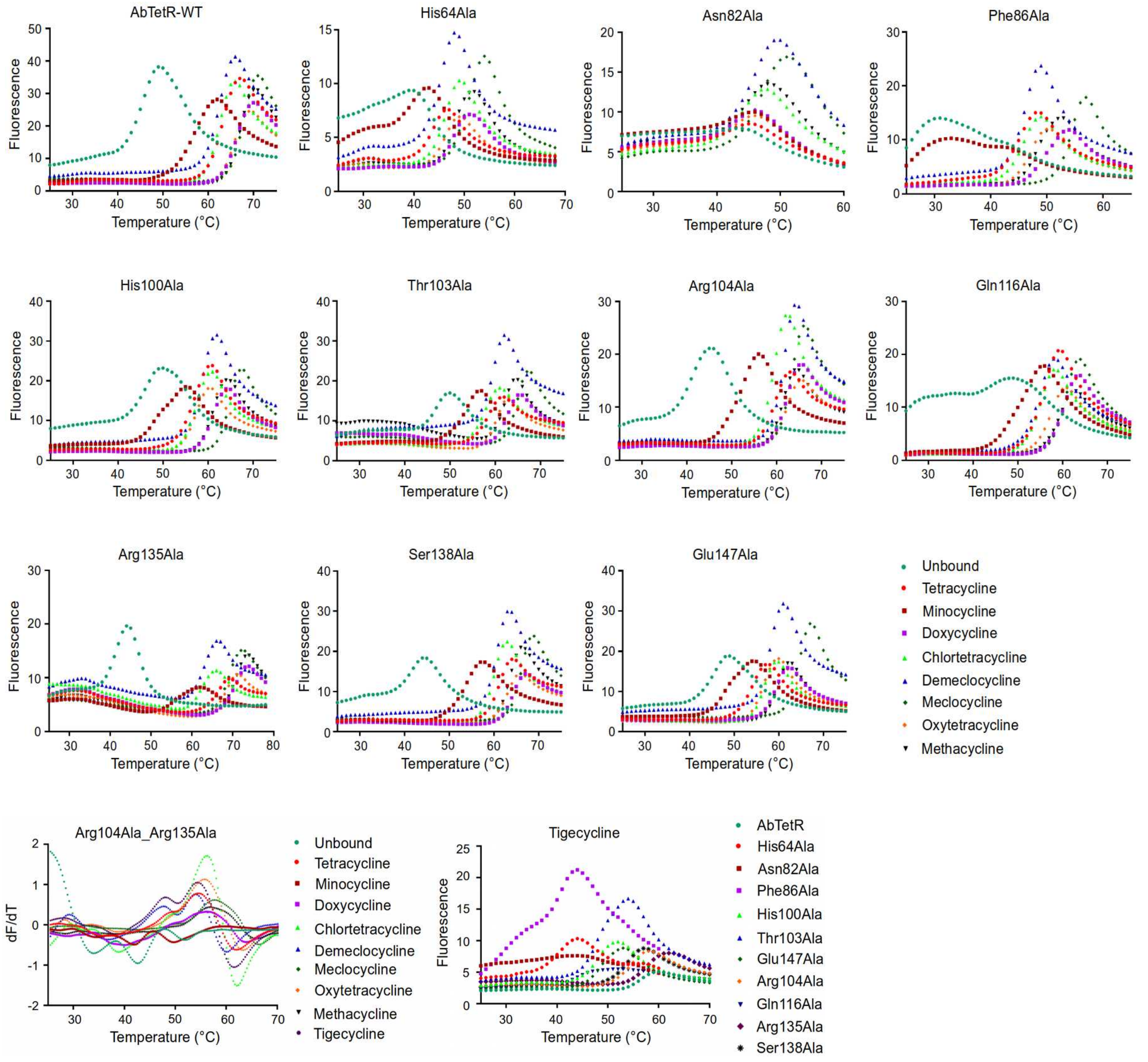

Supplementary Figure 2. Thermal stability of AbTetR and AbTetR substitution variants in the presence of tetracyclines. Experiments were conducted with AbTetR and AbTetR variants in the absence or presence of chlortetracycline, demeclocycline, doxycycline, meclocycline, methacycline, minocycline, oxytetracycline, and tetracycline. SYPRO Orange was used as a reporter to detect the thermal unfolding of AbTetR variants. The resulting fluorescence from SYPRO Orange binding to hydrophobic patches of the unfolding protein was detected at an excitation wavelength of 470 nm and the emission wavelength of 555 nm. Experiments were conducted five times (technical replicates) except for tigecycline and the Arg104Ala-Arg135Ala variant (three replicates) and the results shown are representative.

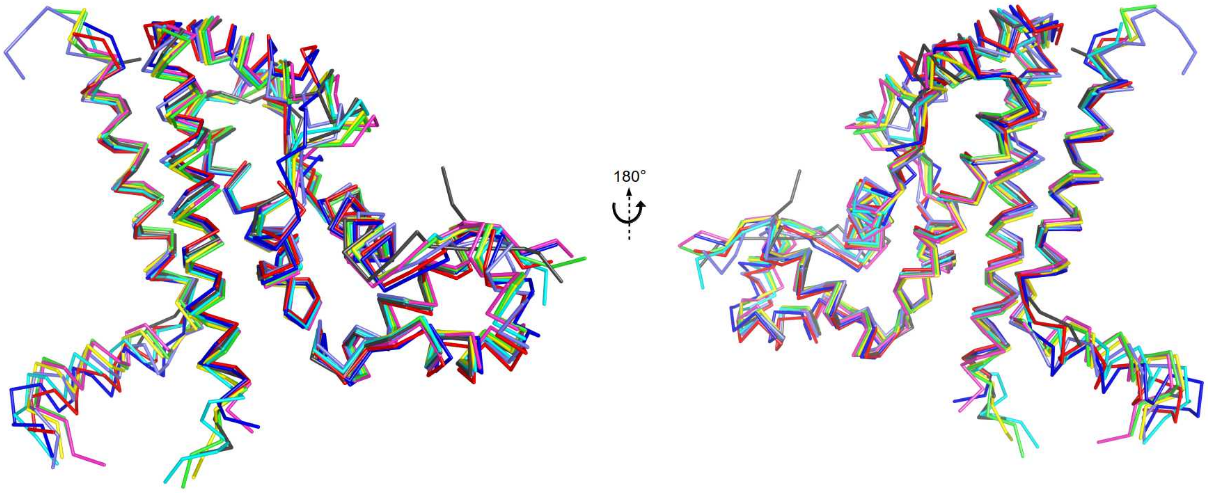

Supplementary Figure 3. Superimposition of the unliganded AbTetR and homologous TetRs. Superimposition of AbTetR monomer A (blue), AbTetR monomer B (red), TetR(A) from *Pseudomonas* sp. (black; PDB: 5MRU), TetR(B) (cyan; PDB: 4AC0), TetR(J) from *Proteus mirabilis* (magenta; PDB: 4D5C), TetR(D) (purple; PDB: 1BJZ, Orth et al., 1999), TetR(H) from *Pasteurella multocida* (green; PDB: 2VPR, Aleksandrov et al., 2008), and TetR(H) from *Mannheimia haemolytica* (yellow; PDB: 4D5F).

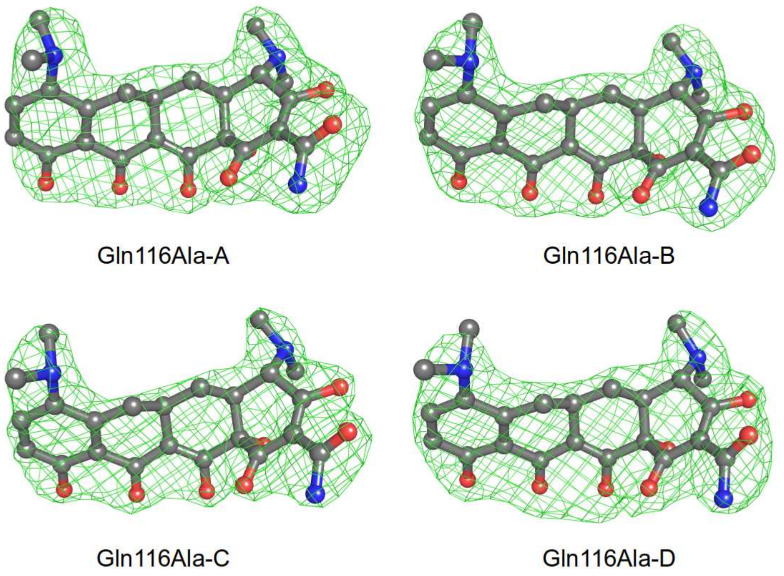

Supplementary Figure 4. Polder electron density map of minocycline bound to Gln116Ala variant. Polder maps are contoured at 4.2 σ for minocycline bound to Gln116Ala-A; 4.8 σ for minocycline bound to Gln116Ala-B; 5.5 σ for minocycline bound to Gln116Ala-C; 4.5 σ for minocycline bound to Gln116Ala-D.

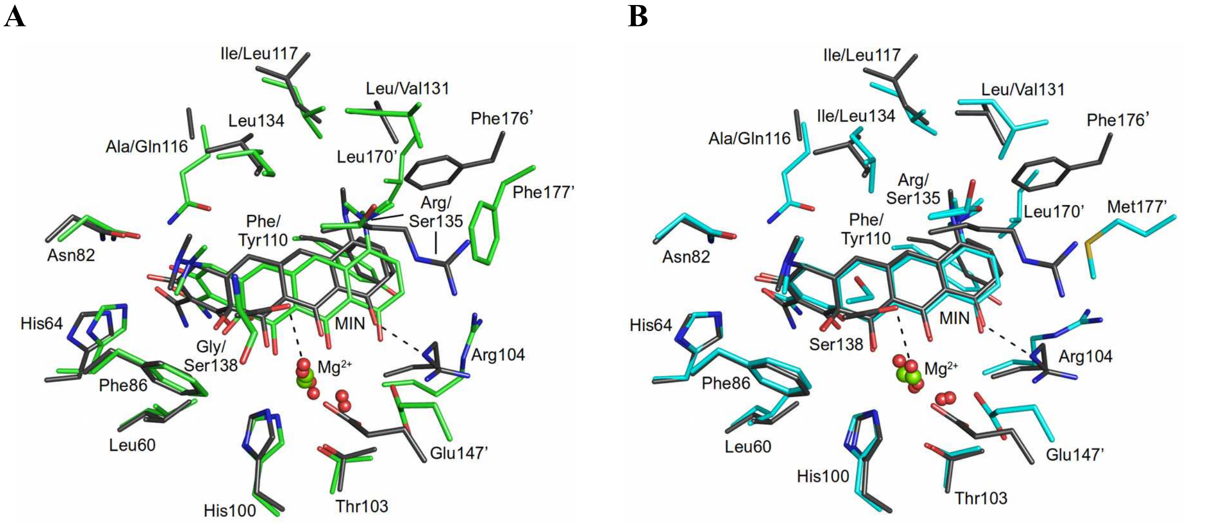

Supplementary Figure 5. Superimposition of the minocycline bound TetRs. AbTetR was superimposed with **(A)** TetR(B) (green, PDB: 4AC0) and **(B)** TetR(D) (cyan, PDB: 2XPV). Minocycline (MIN) and residues of AbTetR are depicted in black. Water molecules are depicted as red spheres.

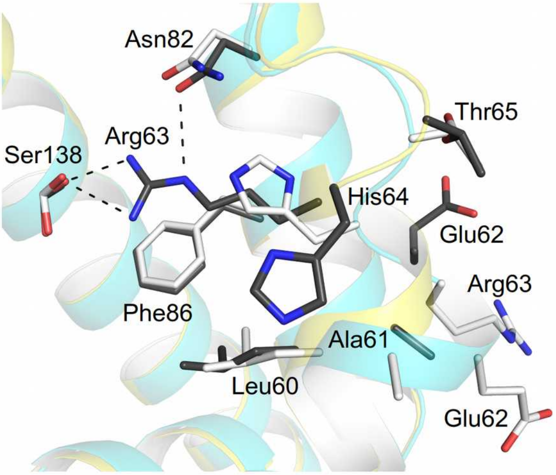

Supplementary Figure 6. Structural differences of the α4 of AbTetR. AbTetR-A and AbTetR-B are shown as yellow and cyan cartoon representation, respectively. Residues of AbTetR-A and AbTetR-B are depicted in black and white stick representation, respectively. Ser138 adopts two alternate conformations.

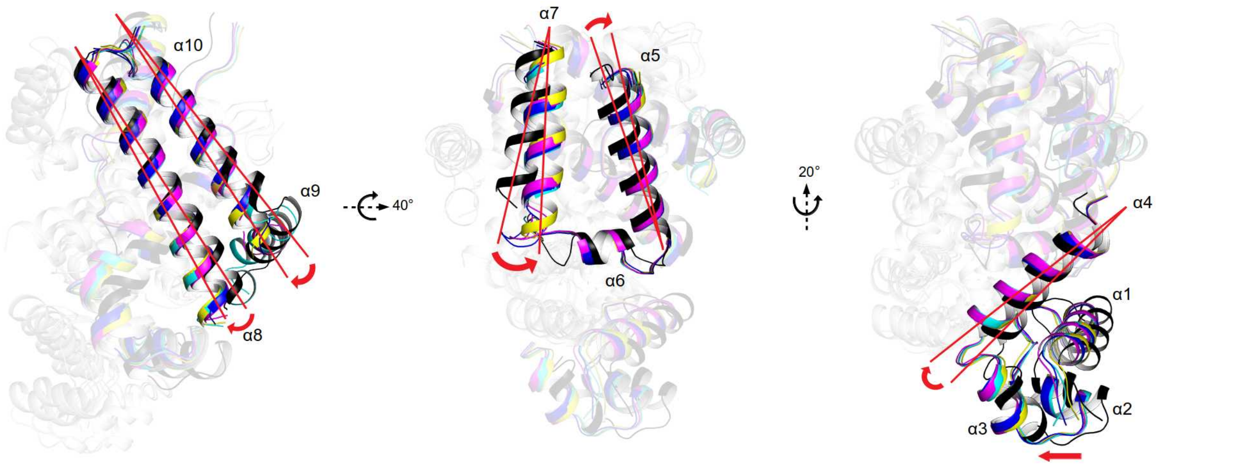

Supplementary Figure 7. Coordination of each of the protomers in AbTetR dimer upon minocycline binding. Minocycline binding to one protomer induces conformational changes of the other protomer in AbTetR dimer. Structures of AbTetR-B, Gln116Ala-A, Gln116Ala-B, Gln116Ala-C, and Gln116Ala-D are depicted as cartoon representation and shown in black, yellow, cyan, blue, and magenta, respectively. Structural superimposition was performed by fixing (superpose) one of the protomer (with main chain atoms) of the unliganded AbTetR dimers and the liganded Gln116Ala dimers. The fixed protomers (subunits) are colored in white. A movement of helices α4/α4', α5/α5', α7/α7', α8/α8', and α10/α10' from each of the protomers in the dimeric protein, in both unliganded and liganded state is depicted with red arrows and lines. A lateral motion of DBD in unliganded and liganded state is depicted with a red arrow.

References

Aleksandrov, A., Schuldt, L., Hinrichs, W., and Simonson, T. (2008). Tet repressor induction by tetracycline: A molecular dynamics, continuum electrostatics, and crystallographic study. *J. Mol. Biol.* 378, 898–912.

Orth, P., Schnappinger, D., Hillen, W., Saenger, W., and Hinrichs, W. (2000). Structural basis of gene regulation by the tetracycline inducible Tet repressor-operator system. *Nat. Struct. Biol.* 7, 215–219.

Supplementary Table 1. Primers sequences.

Plasmids	Primer sequences
pET24AbTetR$_{His}$<br>*tetR* gene	Forward 5'-TTTAAGAAGGAGATATACATATGACCAAACTGGACAAGGGC-3'<br>Reverse 5'-GATCTCAGTGGTGGTGGTGGTGGTGATCTGTTGTAGATGAACGCAGCC-3'
pET24AbTetR$_{His}$<br>Vector	Forward 5'-CACCACCACCACCACCACTGAGATC-3'<br>Reverse 5'-ATGTATATCTCCTTCTTAAAGTTAAACAAAAT-3'
pET24AbTetR-H64A$_{His}$	Forward 5'-GCTACCCGCTCGCTACCCGA-3'<br>Reverse 5'-GCGTTCCGCCAGCATCGC-3'
pET24AbTetR-N82A$_{His}$	Forward 5'-GCTGCCCTGAGCTTCAGAACG-3'<br>Reverse 5'-CTCTTTCAGGAACACCCGC-3'
pET24AbTetR-F86A$_{His}$	Forward 5'-GCCAGAACGGCGTTGCTCTCT-3'<br>Reverse 5'-GCTCAGGGCATTCTCTTTCA-3'
pET24AbTetR-H100A$_{His}$	Forward 5'-GCTGCCGGCACTCGACCGA-3'<br>Reverse 5'-GATACGCGCGCCGTCCCGA-3'
pET24AbTetR-T103A$_{His}$	Forward 5'-GCTCGACCGACAGAACCGA-3'<br>Reverse 5'-GCCGGCATGGATACGCGC-3'
PET24AbTetR-R104A$_{His}$	Forward 5'-GCACCGACAGAACCGAATTTTGG-3'<br>Reverse 5'-AGTGCCGGCATGGATACGC-3'
pET24AbTetR-Q116A$_{His}$	Forward 5'-GCAATACGCTTTCTCTGCGCGG-3'<br>Reverse 5'-CGTCTCGGCGGTGCCAAA-3'
pET24AbTetR-R135A$_{His}$	Forward 5'-GCGGCGGTCAGTCACTATGTG-3'<br>Reverse 5'-GAGCGCCCAAACGGCGCG-3'
pET24AbTetR-S138A$_{His}$	Forward 5'-GGCGGTCGCACACTATGTGG-3'<br>Reverse 5'-CGGAGCGCCCAAACGGC-3'
pET24AbTetR-E147A$_{His}$	Forward 5'-GCTCAGCAGGCATCTGATGCCG-3'<br>Reverse 5'-GAGAACGGAACCGACCACAT-3'
**Operator sequences**	
319 bp	Forward 5'-TGCTGAACCTTGAGGCGTTC-3'<br>Reverse 5'-GCACAAGCTCACGCAGAAGC-3'
216 bp	Forward 5'-TGCTGAACCTTGAGGCGTTC-3'<br>Reverse 5'-CTGAAACTCCAGCGCCGC-3'
206 bp	Forward 5'-TCACGTTCCTTCGCCAGTG-3'<br>Reverse 5'-GCACAAGCTCACGCAGAAGC-3'
103 bp	Forward 5'-TCACGTTCCTTCGCCAGTG-3'<br>Reverse 5'-CTGAAACTCCAGCGCCGC-3'
70 bp	Forward 5'-TCACGTTCCTTCGCCAGTG-3'<br>Reverse 5'-ATCAACGATAAGGAGATGGAC-3'
65 bp	Forward 5'-TAAGTTATATGTCCATCTCCTTATC-3'<br>Reverse 5'-CTGAAACTCCAGCGCCGC-3'
96 bp non-binding sequence	Forward 5'-CGAATCAAGCAGGAACCATC-3'<br>Reverse 5'-TCCGAGTTGCAAGACCGAG-3'

Supplementary Table 2. Data collection and refinement statistics of AbTetR. *Data of the minocycline bound Gln116Ala structure was processed to remove ice-ring artefacts. The high R_{merge} is also attributed to a slight decay in resolution for images 3000-3600.

	**Unliganded AbTetR**	**AbTetR-Gln116Ala in complex with minocycline**
**Data collection**		
PDB	6RX9	6RXB
Space group:	$P2_1$	$P2_12_12_1$
Cell dimensions		
a, b, c (Å)	43.33, 85.11, 62.35	62.54, 64.23, 220.95
α, β, γ (°)	90.00, 109.46, 90.00	90.00, 90.00, 90.00
Resolution (Å)	48.37–1.80 (1.84–1.80)	48.41–2.25 (2.32–2.25)
R_{merge}	0.061 (1.861)	0.256* (1.986)
$I / \sigma I$	20.3 (1.2)	6.0 (1.4)
Completeness	99.7 (99.5)	100.0 (100.0)
Redundancy	13.7 (13.9)	13.7 (14.0)
$CC_{(1/2)}$	0.999 (0.732)	0.991 (0.652)
Refinement		
Resolution (Å)	48.37–1.80	48.41–2.25
No. of reflections	37479	41073
R_{work}/ R_{free}	0.2079 / 0.2369	0.2404 / 0.2760
No. atoms		
Protein	3024	5941
Ligand/ion	5	150
Water	188	128
B-factors		
Protein	46.43	57.38
Ligand/ion	86.33	49.13
Water	48.05	48.70
r.m.s. deviations		
Bond length (Å)	0.0020	0.0015
Bond angles (º)	1.1312	1.1475
